# Supplementary material for: Long-acting injectable antipsychotics for early psychosis: A comprehensive systematic review
Source: PLoS One. 2022 Apr 29;17(4):e0267808. doi: 10.1371/journal.pone.0267808 (PMC9053823; doi:10.1371/journal.pone.0267808)
Supplement: Attachment — Submitted filename: Response to Reviewers.doc [file pone.0267808.s002.doc]

# **T H E U N I V E R S I T Y O F B R I T I S H C O L U M B I A**

|   Dr Kyle J Burghardt Academic Editor PLOS ONE  31 January 2022 |  |
| --- | --- |

Dear Dr. Burghardt,

**Re: Manuscript ID: PONE-D-21-23982**

**Title:** “Long-acting injectable antipsychotic drugs for early psychosis: A comprehensive systematic review.”

Enclosed is our revised version of the manuscript with one copy showing “track changes” as well as a “clean” version.

We are very grateful to the reviewers for their constructive comments and their effort committed to reviewing our manuscript. We feel that the manuscript is now significantly improved.

With best regards,

Alasdair

Alasdair M Barr, Ph.D.

Associate Professor

Department of Anesthesiology, Pharmacology & Therapeutics

2176 Health Sciences Mall, UBC

Vancouver, B.C. Canada V6T 1Z3

Tel: (604) 875-2000 x4728

Fax: (604) 822-6012

Email: [al.barr@ubc.ca](mailto:al.barr@ubc.ca)

Additional Editor Comments (if provided):

Thank you for your patience in reviewing your article. Please see and response to reviewer reports attached. Please revise you manuscript according to these comments and provide a reviewer response table as instructed by PLOS.

Can you please double check reference #34. From my reading, there were 3 groups compared in this study, more LAIs other than paliperidone were included, and the adherence numbers of (25% versus 8.6% adherent; p<0.001) were not listed in #34 reference? Could you please re-check the accuracy of data reported for this reference and others within your report as a double check? Thanks.

WE THANK THE EDITOR FOR NOTING THIS. IT HAS NOW BEEN CORRECTED (LINES 368-376).

Review Comments to the Author

Please use the space provided to explain your answers to the questions above. You may also include additional comments for the author, including concerns about dual publication, research ethics, or publication ethics. (Please upload your review as an attachment if it exceeds 20,000 characters)

Reviewer #1: Line 53: The word “however” doesn’t seem to fit after the previous sentence. Consider removing.

AS THE REVIEWER SUGGESTS, WE HAVE NOW REMOVED THE WORD.

Line 55: Nonadherence rate of 39% seems too specific to one program. Consider finding a more generalized estimate among treatment programs with this same population.

WE HAVE NOW REMOVED THIS SENTENCE, AS (THE REVIEWER NOTES) THERE IS A CONSIDERABLE RANGE AMONG DIFFERENT PROGRAMS WHICH IS HARD TO ESTIMATE FROM THE GENERAL LITERATURE.

Line 56: Consider the word “misuse” in place of “abuse”. This term is considered more stigmatizing and many substance use disorder organizations are urging healthcare professionals to switch the language to be less stigmatizing.

WE HAVE NOW CORRECTED THIS – THANK YOU FOR THE SUGGESTION.

Line 67: Missing the word “to”.

HAVE NOW ADDED IN “TO”.

Line 265: Comma missing after [49].

NOW ADDED.

Line 300: Missing comma and a space after [38].

CORRECTED, THANK YOU.

Line 359: Try to avoid starting a sentence with a number if possible.

THIS HAS NOW BEEN CHANGED TO MERGE WITH THE PRIOR SENTENCE.

Line 388: Try to avoid starting a sentence with a number if possible.

AS ABOVE, CHANGED NOW.

Line 396: A space is needed between “who” and “experienced”.

THIS HAS NOW BEEN CORRECTED.

Line 516: Consider using person-first language such as “patients with schizophrenia”.

THIS HAS BEEN CHANGED AS THE REVIEWER SUGGESTS.

Reviewer #2: Overall this was a well written manuscript.

THANK YOU.

The limitations that I marked while reading through the article were all pointed out in the limitation section of the manuscript. I believe the authors did touch on this, but when working with different definitions of remission, relapse, etc. it can be harder to generalize the results.

WE AGREE – IT IS DEFINITELY A CHALLENGE WHEN CONDUCTING A SYSTEMATIC REVIEW

Some of the studies used DSMIV criteria for the diagnosis however DSM5 was released in 2013 I believe, so I wonder why some of the studies dating in 20019, 2018, still utilized DSMIV

THE REVIEWER NOTES AN IMPORTANT POINT. THE REASONS ARE MULTIPLE (FROM OUR UNDERSTANDING OF THE STUDIES) WHICH POTENTIALLY INCLUDE A TIME LAG FROM WHEN THE STUDY WAS PLANNED UNTIL IT WAS PUBLISHED THROUGH TO A LACK OF UPTAKE OF THE DSM5 FOR RESEARCH STUDIES.

It may have been beneficial to make one of the inclusion criteria "LAIs approved in each country". Because for some of the results Flupentixol (for example) had an advantage but since it is not available in the US, I am unable to utilize that information

WE THANK THE REVIEWER FOR THIS SUGGESTION, AND CERTAINLY SEE THE VALIDITY OF THE POINT. WE HOPE THAT BY USING THE PRESENT APPROACH, WE HAVE EXPANDED THE SCOPE OF THE REVIEW TO BE AS BROAD ACROSS THE INTERNATIONAL COMMUNITY AS POSSIBLE, REFLECTING THE DIVERSE READERSHIP OF PLOS ONE.

Reviewer #3: Thank you for allowing me to review your work. The use of LAIs in early psychosis is an important topic and I believe your study will add to the growing body of evidence to support this practice.

THANK YOU.

Please see below for specific comments:

• Introduction line 61: There have been studies of the economic impact of LAIs that you could cite here which help to address the questions readers may have about the increased cost of LAI formulations compared to OAPs.

WE HAVE NOW ADDRESSED THIS POINT IN LINES 70-74 (IN THE TRACK CHANGES VERSION).

• Introduction line 68: Consider elaborating more on why LAIs are useful in patients with poor adherence for those who may not be as familiar with the medications (i.e. they can be dosed less frequently than OAPs).

THIS HAS NOW BEEN CHANGED AS SUGGESTED.

• Introduction lines 77-79: What is the benefit of including other study types and first-generation agents? (e.g. generalizability, sample size, real-world applicability, etc.)

THIS IS NOW CLARIFIED ON LINES 84-85

• Methods line 83: State here that you included studies that did not compared efficacy of LAI to OAP- the reader may wonder this since it was the focus of your results reported.

THIS HAS BEEN CLARIFIED ONLINE 90.

• Table 1: Given the size of the table, consider writing out some lesser-known abbreviations (e.g. OL = open-label, NS = not specified, DB = double-blind?, DUP = duration untreated psychosis?) since there is space to do this. It saves the reader from having to scroll up and down several pages each time they need to look up an abbreviation

THIS HAS BEEN DONE.

.
• Table 1: The Dubois 2014 article describes e-STAR and TIMORES groups- these acronyms will need to be spelled out in the footnote and/or some indication given of how the 2 groups differed for the table entry to make sense to the reader.

THIS HAS BEEN DONE.

• Table 1: Convert all study durations to months for consistency and ease of comparison.

AS SUGGESTED, THIS HAS BEEN DONE.

• Table 1: Be consistent with how age is reported (e.g. sometimes it is median, sometimes range is included, Ruan 2010 has +/- for SD instead of (SD)) and if possible, calculate mean (SD) for all.

THIS HAS BEEN DONE WHERE POSSIBLE, BUT SOME STUDIES ONLY INCLUDE THE MEDIAN AGE.

• Results: Include the follow up period for discontinuation and remission wherever possible.

AS SUGGESTED.

• Results: Include measures of statistical significant wherever possible when comparing outcomes between LAI and OAP groups.

THIS HAS NOW BEEN DONE.

• Results line 150: Define LOCF- not sure what this endpoint is and if this study is comparing LAI vs. OAP like the others.

THIS HAS NOW BEEN DEFINED. IT IS A POST-HOC ANALYSIS OF A RANDOMIZED, DOUBLE-BLIND 52-WEEK STUDY EVALUATING EFFECTS OF 25 MG OR 50 MG RLAI IN PATIENTS WITH SCHIZOPHRENIA AND SCHIZOAFFECTIVE DISORDER. DATA WAS STRATIFIED USING TIME SINCE DIAGNOSIS (≤ 3 YEARS AND > 3 YEARS). USED LAST-OBSERVATION-CARRIED-FORWARD (LOCF) METHODOLOGY FOR END POINT ANALYSIS.

• Results line 156: This study needs to be explained more clearly- what made the e-STAR and TIMORES groups different that accounted for the change observed in CGI-S?

THIS HAS BEEN CLARIFIED ON LINES 166-173

• Results line 159: Is this the LAI group specifically? How did it compare to the OAP group?

THIS HAS NOW BEEN CLARIFIED

• Results lines 162-175: It would be helpful to remind the reader that these were studies with no comparator group, so you are only reporting the outcomes for the LAI (unlike the studies previously described in this section). The description of “open-label” is not sufficient for the reader to know this as many RCTs are also open label.

THIS HAS BEEN NOTED (LINES 192-193).

• Results lines 306-307: This statement is an interpretation of the results and should be in the discussion section.

THIS SENTENCE HAS NOW BEEN REMOVED FROM THE RESULTS. IT DIDN’T FIT WELL INTO OUR REVISED DISCUSSION SECTION, SO WE HAVE JUST EXCLUDED IT.

• Results lines 336-337: Define IQR.

THIS HAS BEEN DONE.

• Results lines 351-354: It may be helpful to mention again that these patients were switched from OAP to LAI PP and that these rates are on LAI.

• Results lines 360-361: Remind the reader that Bossie compares recent vs. chronic and Cervone is PP and has no comparison group. It is unclear as currently which groups you are referring to for the discontinuation rates reported.

THIS HAS BEEN CLARIFIED

• Results lines 405-407: These statements belong in the discussion section.

WE HAVE REMOVED THESE STATEMENTS.

• Results line 439: Describe the MATRICS MCCB assessment.

THIS HAS BEEN DESCRIBED.

• Results lines 462-465: Not sure how these results are relevant to your study aims. I would suggest leaving them out.

THIS HAS NOW BEEN REMOVED.

• Results line 489: Define GAF.

THIS HAS BEEN DONE.

• Results lines 489-491: This statement belongs in the discussion section.

WITH OUR EXTENSIVELY REVISED DISCUSSION, THERE WAS NO OBVIOUS PLACE TO INCLUDE THIS ONE POINT. WE HAVE THEREFORE TAKEN THE LIBERTY OF KEEPING IT IN THE CURRENT PLACE (WE FEEL THE POINT HAS VALUE, AS IT WAS A SUMMARY POINT OF THE AUTHORS) BUT WILL READILY REMOVE IT IF THE REVIEWER DISAGREES WITH US.

• Results lines 508-510: This statement belongs in the discussion section.

THIS HAS BEEN DONE.

• Discussion: This section needs to be improved. Most of the discussion is devoted to the findings of other reviews. It appears that your main conclusion from your study is that “Overall, it seems that LAIs are effective pharmacological treatments and may reduce the risk of relapse in early psychosis patients.” This is something that your review can add support for but is rather weak based on your findings alone since there was no meta-analysis performed. It appears that your aim was to include a wider range of trial designs for a more “comprehensive” review on this topic, which I believe is a strength that makes your study unique. I would go on to explain more about why this is important and why we need more reviews that include naturalistic study designs to assess real-world utility of LAIs. However, you go on to conclude that there is a need for more RCTs- will these be as useful for demonstrating the benefit of LAIs? See the following studies:
o Haddad PM, Taylor M, Niaz OS. First-generation antipsychotic long-acting injections v. oral antipsychotics in schizophrenia: systematic review of randomised controlled trials and observational studies. Br J Psychiatry Suppl. 2009 Nov;52:S20-8. doi: 10.1192/bjp.195.52.s20. PMID: 19880913.
o Biagi E, Capuzzi E, Colmegna F, Mascarini A, Brambilla G, Ornaghi A, Santambrogio J, Clerici M. Long-Acting Injectable Antipsychotics in Schizophrenia: Literature Review and Practical Perspective, with a Focus on Aripiprazole Once-Monthly. Adv Ther. 2017 May;34(5):1036-1048. doi: 10.1007/s12325-017-0507-x. Epub 2017 Apr 5. PMID: 28382557; PMCID: PMC5427126.

WE HAVE MADE EXTENSIVE REVISIONS TO THE DISCUSSION TO ADDRESS THE POINTS RAISED BY THE REVIEWER. WE ALSO NOW INLCUDE THE HADDAD REFERENCE.

• Limitations: Discuss the issues with including studies from different countries (i.e. practice patterns/guidelines, cost/insurance models, utilization rates, etc.).

THIS IS NOW ADDRESSED ON LINES 645-646
